# Perspective in Paediatrics
*Delivered to the West of England Child Health Group, December 7th, 1954.


**Published:** 1956-04

**Authors:** W. S. Craig

**Affiliations:** Department of Paediatrics and Child Health, University of Leeds


### PERSPECTIVE IN PAEDIATRICS*

BY

PROFESSOR W. S. CRAIG

*Department of Paediatrics and Child Health, University of Leeds*

I have often heard from Professor Neale of the many activities of your flourishing West of England Child Health Group. It was not however until I came to prepare my notes for this evening that I realized the difficulty of presenting a subject in a way likely to appeal to members sharing such a wide variety of interests in child health. The problem was to preserve a sense of perspective. Then it struck me—is preservation of perspective not a problem with which all of us are concerned? Hence the choice of subject for this evening.

### UNIVERSITY DEPARTMENTS

There is I think hesitant perspective in the names given to university departments up and down the country. " Paediatrics " is out of fashion. It lacks reference to preventive considerations. None the less—derived as it is from the Greek word meaning therapeutics of infancy it has the merit of reminding us that, no matter the advances of what has been termed positive medicine, the need for treatment and cure will always remain. " Child Life and Health " appeals to me because of its associations with Edinburgh and because of its implied philosophy—but even so, it is not wholly satisfying. " Child Health " has the attraction of brevity and directness and puts paid to at least one perennial etymological joke; nevertheless it involves risks of over-simplification and encourages an attitude of mind which culminates in regarding those interested in the care of the child, whether in health or disease, as " baby doctors ".

### IS PAEDIATRICS A " SPECIALITY "?

The term " Speciality " is not disparaging, but it implies specialization of the narrowest kind. This is entirely contrary to the interests of the children for whose health we are responsible. Indeed I would suggest that the time has come when we should exercise care in depicting the study and practice of child health as a speciality. I say this because the terms speciality and specialization have acquired a new significance of recent years. " Specialization " implies a high degree of skill and knowledge in relation to a group of diseases or conditions with a common anatomical, functional or clinical basis.

It may be argued that Child health is a speciality because it is concerned with a particular age range. But to say this is to ignore that childhood in the sense *we* know it is incapable of precise chronological definition. Would any of us venture to say at what stage in embryonic development ante-natal paediatrics begins? Or again who would deny that we have a contribution to make to post-adolescent follow-up care in the form of vocational guidance?

Furthermore, the expansion of child health as a study and practice has taken place on many planes. There is convincing evidence of this in the presence here tonight of family doctors, health visitors, district nurses and midwives. Child health knows no boundary separating domiciliary from institutional care. None the less underlying all paediatric activities is recognition of the home as the dominant factor in child life, and services intended for the care of child life must be based on the home and not on the hospital. Consider for a moment the services that exist—for

* Delivered to the West of England Child Health Group, December 7th, 1954.

the healthy, the ailing, the handicapped, the deprived and the delinquent child. Schools and clinics of many kinds, nurseries, convalescent homes, hospitals, children's courts, remand homes—all are involved. Nor should we forget voluntary organizations—Dr. Barnardo's Homes and The National Children's Home; youth organizations such as the Boy Scouts and Girl Guides associations, the Boys' Brigade, and the many boys' and girls' clubs; the St. John Ambulance, the British Red Cross and Women's Voluntary Service. Each in their own way supplements the statutory services administered through welfare, school health, children's and mental health committees. None of us can afford to ignore the work of these numerous aspects of child health.

That being so, and to return to my original point, can we afford to perpetuate the conception of child health as a specialist study in the modern sense of the term? I think not, if the *total* work of those engaged in the care of child life is to be presented in its true perspective to our professional colleagues and to the general public.

So much, then, for perspective in so far as it concerns the relations of child health on the one hand and general medicine and general sociology on the other. And now, what of a sense of perspective within our own ranks? When I say in our ranks I have in mind all—and " all " without reservation—all concerned with the care of child life whether in home or foster-home; in hospital or school; in voluntary, official professional or lay capacity.

### SPECIAL INTERESTS

If we are honest we must admit that the subject we have in common and at heart is always liable to a certain risk of fragmentation because we each have our particular interests. We find difficulty in seeing the work of others in proper perspective and we often fail to see ourselves as others see us. The paediatrician is no exception. For too long too many of us have allowed hospital considerations to restrict our studies.

True, there has been a change of recent years. An active *entente cordiale* links us with the infant welfare and school health services. But the recently published survey, *The Thousand Families in Newcastle upon Tyne*, reminds us that our perspective is still in need of adjustment. We have still to learn how and what to teach the medical student. To do this we can learn from the practising family doctor. The late Sir James Spence and his collaborators made a plea for what they call " a continuing process of mutual education " between specialist and family doctor in order that they may be the " better equipped for the care of children".

### DOMICILIARY AND INSTITUTIONAL CARE

I feel sure that this process of mutual education is one of the objectives of your Group. It is a principle which I should like to see extended far and wide, and expanded to include all workers engaged in the care of child life. I am convinced the activities of midwives and health visitors would benefit were they kept better informed of advances in each other's work. I am equally certain that the skill of the trained sick children's nurse in hospital would be enhanced had she regular opportunities to study the work of the health visitor and district nurse on the field. The reverse is equally true. Were a district nurse or health visitor to come into hospital out-patients departments *and* hospital wards at regular intervals, her work on the field would benefit from increased understanding of what her colleagues in child care are doing. A similar approach would do much to solve prevailing misunderstandings between the almoner and health visitor.

I would urge that there is a need for periodic interchange of workers on the field with those in institutions. This implies interchange among employees of different statutory bodies. It may well be that disturbing administrative adjustments would be required—but a nicely balanced smoothly running administration is not efficient which curbs the professional efficiency of its officers, and which prefers that the child shall adapt itself to the administrative framework and not the framework to the child.

### SOME MISUNDERSTANDINGS

There are many misunderstandings the causes of which are to be found in inherited antagonism and in the dictates of theoretically wise economy. The relations between voluntary and municipal effort often lack harmony to the detriment of the child. And there is no denying that the relationships between the family doctor and his colleagues in the environmental services are not everywhere what they might be. Of course, personalities offer part of the explanation—but only a very small part. Fundamentally we are still suffering from inability to see paediatric history in perspective. Is it not time that once and for all voluntary and municipal effort should recognize and accept their mutual indebtedness? Many a children's voluntary organization today owes its own survival and survival of its ideals to public money; and many a local authority service for children traces its origin and inspiration to private endeavour. Then, too, there is the family doctor who resents the intrusion of the welfare services—but, is it really this that he resents? Is he not perhaps keeping a rather tattered flag flying? And do infant welfare and school medical officers not rather often anticipate antagonism where there is no need? I well remember my father's chagrin on his return from the First World War to find infants in his practice attending a previously undreamt of child welfare clinic. The care of children had always been one of his main interests and the finding came as a shock to his professional pride—but he soon accepted the situation with a smilingly good grace which will always remain a lesson to me.

### PERSPECTIVE IN ECONOMY

Turning to economics, sound or unsound—new proposals often fail to secure the necessary support on the grounds that they are too narrowly specialist in conception. None the less the net result of insistence on economy in one speciality is sometimes to facilitate prodigal expenditure in another. Economy is a virtue in those responsible for the distribution of funds whether public or private. But surely control of expenditure must have a positive as well as a purely negative purpose. Poor Law relief was once described as a salve to the public conscience. Be that as it may, execution of the Poor Law was sometimes and in some places concerned primarily with conserving national income and only secondarily with the promotion of improved social conditions. This attitude should have no place today.

True perspective in economy requires two things. The first—less attention to the relative merits of this or that interest, and more consideration of the total needs of the child community; and the second—if we are sincere in our endeavours for children, greater thought for generations as yet unborn, indeed, as yet not conceived.

I should like to illustrate my points if I may. You know of several international schemes furthering large-scale efforts on behalf of children in the so-called economically backward countries. I have no first-hand knowledge of these, but talking with those who have, I have been impressed by the anxiety expressed lest measures on behalf of mothers and children in these countries should be developed independently of other measures directed towards the advancement of general medical and general public health services. Put another way, concentration on child welfare is only partially effective if such co-existing problems as those of epidemiology, sanitation, nutrition, husbandry and even stock-rearing do not receive equal and simultaneous attention.

You may say that we in this country do not know the ravages of malaria and worm infestation; we have, comparatively speaking, a safe water supply and sound measures of sewage disposal; and we import what we lack in the way of meat and grain. This may well be so; but whether it be in Europe or in Eastern Asia the requirements of maternal and child welfare are but one facet, in the needs of national welfare.

And to particularize—I would say that, the world over, child welfare has in common the problem of housing no matter whether the unit of housing be a croft, a basement room, a tenement dwelling, a mud hut, wigwam or kraal. My subject is perspective in paediatrics. When we recommend expenditure on this and that refinement of the hospital and clinic care of children, we should be mindful at least of the extent to which our demands may interfere with remedying the greatest single source of the many clinical and social problems with which we have to deal—housing.

COMPLACENCY CONCERNING ENVIRONMENT AND HOUSING

If perspective is vital to successful paediatric care—and I think it is urgently so, then collectively we should spare no effort in making accelerated large-scale improvement of housing conditions the spearhead of progress in child care. In saying this I am not ignoring the substantiated claims of successive governments in this connexion. But I ask you—think of the worst homes from which children come to you for guidance and help. If *you* had been born into such circumstances, or if *you* had had to bring up a family in such surroundings—what would *your* reactions have been? What effect would environment have had on *your* development as an independent citizen? We accept our own good fortune, and the misfortune and mischances of others far too complacently. Indeed no matter the position we hold, our good fortune—whether in the matter of birth or career seriously handicaps our sincerest endeavours to understand the mischances of our fellow human beings.

We still have a long way to go to give effect to the words of the American philosopher John Dewey (1859–1952):

" What the best and wisest parent wants for his own child that must the community want for all its children."

In the present century paediatric care has experienced many thrills—the virtual disappearance of deficiency diseases, scarcely believable advances in therapy, traditional beliefs dispelled and rare unsuspected conditions revealed by new biochemical methods, modernized approaches to physical and mental handicap, and frank recognition of deprivation in childhood. But whatever the thrill of individual advances they must be seen in relation to the total needs of child care.

I spoke of deprivation in childhood. The Children Act is concerned with children deprived of normal home life. These children are considerable in number but their number is as nothing compared with those who know only such of home life as their inadequate and overcrowded housing conditions permit. Surely the problem demands greater urgency of insistence on the part of those devoted to the care of child life.

In other countries schemes to counter deaths from prematurity have been criticized because they ignore the probability of death from starvation in later childhood. It is argued that perspective is lacking. Measures for the promotion of health in childhood in this country are equally lacking when they fail to urge the paramount need for housing to have priority consideration.

But the problem does not end there. Child health—physical, emotional and mental—cannot be considered apart from family health. Already geriatric problems in an ageing population are having their repercussions on domestic and community aspects of child life. Measures for the care of child life must be developed with an eye to the future when the force of national economic circumstances may well deprive child care of much of the favoured consideration it now rightly receives.

Planning must be realistic. Effective child care is dependent in the last resort on sustained national well-being, and national well-being can brook no waste of resources whether of finance or woman power. And I would suggest that the one certain way to ensure long-term—and perspective in paediatrics must have regard to long-term considerations—the one certain way to ensure long-term economy is to concentrate *now* on housing—even if this involves curtailment of ambitious hospital and clinical extensions for children. I would even add *ambitious* school extensions.

I am afraid I am in danger of giving the impression that perspective in paediatrics is an entirely domiciliary problem. This is far from being the case. If I am honest I must admit that I have left hospital considerations to the last because they leave me more bewildered than any others. Perhaps it is that near perspective is less precise than distant perspective.

### HOUSE PHYSICIAN AND REGISTRAR

And the first of these problems is the position of the paediatric registrar and the house physician. Frankly my sympathies are with the house physician. In the early years of my training there were no registrars and I received a grounding from my close friend and former chief Professor Charles McNeil which, even had I his sagacity and ability, I could not give to my house physicians today. This is the more unfortunate because today the clinical material in the wards is less typical than it used to be of the general run of domiciliary diseases in childhood; and because, whatever the individual student's ultimate objective may be, his first aim should be to acquire knowledgeable experience of general practice.

I am convinced that future preventive and curative domiciliary care would derive immense benefit were apprenticeship to a family doctor required as part of the pre-registration period. I served no conventional apprenticeship—but, as the son of a family doctor, I grew up in an atmosphere of general practice, and in reality my apprenticeship started unbeknown to my father or myself long before my student days. With each year that passes I realize the irreplaceable value of that unconscious introduction to medicine in general and to child health in particular.

The problem is how to convey something of that to the house physician. For help we should turn to the registrar—he holds a key position. If he goes the wrong way about things he can lead the recent graduate by way of the laboratory and the x-ray department to a conception of disease in childhood epitomised in the tabulated transactions of learned societies. On the other hand I know of no greater source of strength to a department than a registrar who despite his own mastery of skilled techniques and laboratory data, normal and abnormal, helps the young house physician to master basic physical signs; to consider physical signs in terms of diagnosis and prognosis; to assess clinical findings, etiology and after-care in relation to a background of home; and then, and only then, to understand where and how laboratory and other aids can and should be utilized to confirm or supplement clinical deductions. In other words perspective should form an essential part of the instruction given the house physician by the registrar—the registrar and the house physician benefit each in their own way, and by studying and watching the process of mutual instruction I invariably learn even more than they do.

### THE NURSE AS CLINICAL COLLEAGUE

And then there is the position of our nursing colleagues—whatever the dependence of the surgeon on his operating theatre sister we owe infinitely more to those who help us in hospital and the home—as ward and out-patient sister, district and home nurse, health visitor and midwife. From whom did we learn the elements of infant feeding? Who is it who so frequently makes the subtle clinical observations which prompt early recognition of post-natal intracranial damage, of urinary infection, of pneumonia or of commencing gastro-enteritis?

The answer to all these questions—is sometimes the mother; more usually; our nursing colleagues. Not only do they carry out the treatment we recommend, but in addition to giving continuous skilled nursing care they help us with diagnosis, with the evaluation of progress and with the planning of subsequent care. In an age when laboratory medicine is in the ascendant, we would do well in our own interests to remember how little we have acknowledged and how much we still depend upon the clinical observations of the skilled nurse. I require it of my house physicians that they include in their notes the salient observations made by the sister—credit being given where credit is due. In this way I aim at encouraging the future family doctor into a way of clinical approach and thought which both accepts and expects professional partnership with the nurse in domiciliary practice.

To return to the hospital ward sister for a moment—much as we value her for clinical help we should remember how much we depend upon her for the efficient administration of our children's wards. The National Health Service has brought about many changes in hospitals. One thing it has not altered. It continues to take for granted the selfless devotion of the ward sister without which no children's ward can ever function effectively. This is an age-long tradition; but I do suggest the time has come when we as professional colleagues of these sisters should ensure that management committees see the demands made upon ward sisters in proper perspective. I am thinking less of children's hospitals than children's wards in general hospitals. In certain general hospitals nursing duties are being undertaken to an increasing extent by assistant nurses, orderlies and even cadets, a number of whom are engaged on a part-time basis, and who are never required to be on duty at the week-ends. The same applies to domestic staff. I know the administrative difficulties—but is it right that demands of orderlies, cadets and domestic staff should be met by ward sisters giving up their off-duty time? This cannot go on indefinitely, and should a major crisis arise hospital care of the child will experience an irretrievable set-back.

### THE OBSTETRICIAN AND MIDWIFE AS BENEFACTOR

I have spoken of clinical medicine. I wonder if those of us who practice paediatrics, and in particular those of us who have the privilege of teaching the subject, always realize our good fortune in being allowed to look after the newly born. No other clinical material offers so numerous and so unexpected challenges to our clinical acumen. At the risk of betraying lack of perspective I must confess to knowing no greater clinical thrill than in assessing clinical progress in an ill newborn child with the object of giving a sound, honest, tentative prognosis while diagnosis is yet uncertain. And where else are there such opportunities for studying health and disease side by side, and for acquiring insight into the mutual ties which bind mother and baby?

I spoke of our good fortune in being *allowed* to look after the newly born. I *meant* " allowed "—because the more enthusiastic the physician, the more liable is he to forget his debt to the obstetrician. It is very sobering to read paediatric history and to learn how much medical care of the child no less than of the infant owed to the obstetrician in the eighteenth and nineteenth centuries. For my part I feel that as paediatricians we must at all costs avoid possessiveness when taking advantage of the free access given us to the neonate.

Paediatric care, if it is to be true to its declared principles must foster the conception of inseparability of mother and child. This to my mind is only possible if the rôle of the paediatrician is to help the obstetrician in his care of the infant on behalf of the mother. You may say " But surely this is always the case ". Certainly, that is the position in domiciliary practice and in maternity hospitals where there are no special baby units. None the less there is always the danger in the teaching hospital that zeal in care of the baby may lead to forgetfulness of the obstetrician—and an even less pardonable crime, forgetfullness of the mother.

A further consideration in the teaching hospital is that the paediatrician undertaking care of the babies must be prepared to teach not only the medical student, the pupil midwife, and the paediatric staff, but also the junior obstetrical staff, many of whom will eventually leave to take up more senior appointments where they will have to undertake the emergency and more immediate care of the newly born infant. I stress this because I know full well I myself was slow to realize how essential it is in the interests of infant care in general, that instruction in specialized management of the neonate must be freely extended without let or hindrance to the obstetrical house physicians and registrars.

If I was slow to acquire perspective in that connexion, I was less slow in another direction. I had just completed a first draft of what I thought was a rather satisfying paper based on ten years' work. My usual practice is to pass such drafts to a registrar for criticism. On this occasion the comment I got was " Yes Sir! It's all right but I'm afraid it shows no knowledge of neonatal care in the labour ward! " And he was perfectly right! My study of the neonate commenced in the days when having made sure the honoraries had completed their rounds one used to slip timidly in an out of the lying-in wards while the ward sisters were at tea. The labour ward never entered into my ken—one might as well have tried to seek entry to a sacred temple. Today things are very different and the paediatric registrar of today should realize his indebtedness to the obstetrician and the midwife for his improved perspective in neonatal paediatrics.

### CLINICAL PERPLEXITIES IN RETROSPECT

There are many other clinical problems in which what I can only describe as emotional considerations make for difficulty in preserving a sense of perspective. There is for instance the mongol baby of a few days old who develops signs of acute intestinal obstruction. Or the spastic microcephalic infant who has survived his first birthday and develops a fulminating descending respiratory infection. What should be done in these circumstances? Should modern skill and therapy be used to prolong severely handicapped lives the span of which used to be limited in the natural course of events? Many factors call for consideration—not least of which are our moral and religious convictions. I do not know what the answer is. It is not as if the answer were for oneself—it is for another.

And yet there is the other side to the question. In the early days of streptomycin treatment of tuberculous meningitis I frequently found myself asking the question: Am I really entitled to pursue treatment associated with scant hopes of survival and scantier hopes of a gratifying end-result and with inevitably prolonged harrowing distress to patient and parents? Even when, with advances in treatment, the prospects of good results improved considerably it was not easy to embark on a course of treatment involving the daily lumbar puncture over months of, even the bravest of children. I can still hear the little girl being prepared by the kindliest of sisters for lumbar puncture crying plaintively:

" No today, tomowwow Sister. I winna cry tomowwow, Sister."

I often wondered if the right decision had been made. Today I have no doubt. At this moment I have under my care four children who, earlier in the year, were admitted to hospital with tuberculous meningitis and who are now running about to all outward appearances healthy and happy—none of them having been lumbar punctured more than two or three times, and then only for diagnostic or follow-up purposes.

Perhaps I am foolishly sensitive, but I confess to never before having known such a sense of relief, gratification—call it what you will, that such a merciful advance in treatment should have become a practical reality. I have had to adjust my perspective—and I realize that today's relief stems from the distress and the courage of the patients and parents of those earlier but not very distant days.

### NATIONAL GOOD FORTUNE IN AN UNCERTAIN WORLD

My time is far advanced. The subject I have chosen is limitless. There are a dozen-and-one important aspects which I have not touched upon—all of which is evidence that in choosing my subject I lacked the very thing for which I am pleading! There is the long, arduous, working day which is imposed on many children by ambitious parents and which often exceeds in length that of the labourer, craftsman or office-worker. There is the problem of infant feeding management with its many variables—the child, the mother, the food and the too readily forgotten, most variable of all variables, the skill of the professional attendant. And there are the unpredictable reactions to education for child health on the wireless—reactions which sometimes betray how easily errors in perspective in medical matters can be conveyed to the laity. I must desist—however! BUT—if I may use the word " perspective " for the last time, may I ask you to see our good fortune in this country in its proper perspective? We cannot afford always to see things through rose-tinted glasses because children in these islands receive care than which there is no better in the world.

In our own Continent of Europe you have not far to go to see nutritional anaemia, and active rickets of a severity and in numbers that have not been seen in this country in the present century. Travel west and, I am told, there you will find cities famed for their pioneer scientific paediatric research situated amidst vast rural populations where child care receives little organized attention. Visit the east, and there there are poverty, ignorance, malnutrition, starvation, affecting not tens and hundreds, but thousands and hundreds of thousands of children.

It always strikes me as strange that in a world in which countless deaths among children still result from poverty and starvation, in a world in which only the very few are tolerably housed—it should be thought worth while to devote so much time and money to research into the study of the causes of delinquency. There is no denying the truth of Joseph Joubert's (1754–1824) dictum of over 150 years ago:

" Children have more need of models than of critics."

One of the most recent studies was concerned with the behaviour problems of children born while a world war was in progress. Is the delinquent child any more irrational in his response to surroundings than the vagrant pauper child of two centuries ago; or any more irrational than the adult of the present century, who knowing want, sees no delinquency in resorting to aggression and war?

We should indeed be thankful that an Indian representative of U.N.I.C.E.F. could recently broadcast:

" Whatever nations think of each other, they usually wish well for each others children."

### CHILD HEALTH—A COMMONWEALTH OF INTERESTS

Child health cannot be viewed separately from the wide considerations of national well-being. Nor can the health of any one nation be considered as something apart from the health of mankind as a whole. The world population increases as food resources decline, and population increases more rapidly where poverty is most prevalent and food resources are least adequate. Paediatrics must be considered against this background. Failure to do so is to ignore global influences which will without question one day have the most far-reaching repercussions on your work and mine; on the work of those who follow us; on the care of child life in this country; and on the standards of child health throughout the world.

For this reason I should like to feel that to-night I have left with you a conception of paediatrics as a Commonwealth of Interests in the Care of Child Life. The term Commonwealth will always be associated with the name of that great statesman the late Field-Marshal Smuts. Let me, then in conclusion remind you of these words of his:

" This Commonwealth is peculiarly constituted. It is scattered over the whole world. It is not a compact territory and it is dependent for its very existence on world wide communications—communications which must be maintained."

